# Real life experience with the wearable cardioverter-defibrillator in an international multicenter Registry

**DOI:** 10.1038/s41598-022-06007-y

**Published:** 2022-02-25

**Authors:** Ibrahim El-Battrawy, Boldizsar Kovacs, Tobias C. Dreher, Norbert Klein, Stephanie Rosenkaimer, Susanne Röger, Jürgen Kuschyk, Ardan Muammer Saguner, Jacqueline Kowitz, Julia W. Erath, Firat Duru, Ibrahim Akin

**Affiliations:** 1 University of Mannheim, Mannheim, Germany; 2 Bergmannsheil University Medical Center, Ruhr University Bochum, Bochum, Germany; 3 Department of Cardiology, University Heart Center, University Hospital Zurich, Zurich, Switzerland; 4 Department of Cardiology, Angiology and Internal Intensive-Care Medicine, Klinikum St. Georg gGmbH Leipzig, Delitzscher Straße 141, 04129 Leipzig, Germany; 5 Department of Cardiology, Frankfurt University Hospital, Goethe University, Frankfurt am Main, Germany; 6 Department of Cardiology and Angiology, Bergmannsheil University Hospitals, Ruhr University of Bochum, Bochum, Germany

**Keywords:** Ultrasonography, Ventricular fibrillation, Ventricular tachycardia

## Abstract

Patients at high risk for sudden cardiac death (SCD) may benefit from wearable cardioverter defibrillators (WCD) by avoiding immediate implantable cardioverter defibrillator (ICD) implantation. Different factors play an important role including patient selection, compliance and optimal drug treatment. We aimed to present real world data from 4 centers from Germany and Switzerland. Between 04/2012 and 03/2019, 708 patients were included in this registry. Patients were followed up over a mean time of 28 ± 35.5 months. Outcome data including gender differences and different etiologies of cardiomyopathy were analyzed. Out of 708 patients (81.8% males, mean age 61.0 ± 14.6), 44.6% of patients had non-ischemic cardiomyopathy, 39.8% ischemic cardiomyopathy, 7.9% myocarditis, 5.4% prior need for ICD explantation and 2.1% channelopathy. The mean wear time of WCD was 21.2 ± 4.3 h per day. In 46% of patients, left ventricular ejection fraction (LVEF) was > 35% during follow-up. The younger the patient was, the higher the LVEF and the lower the wear hours per day were. The total shock rate during follow-up was 2.7%. Whereas an appropriate WCD shock was documented in 16 patients (2.2%), 3 patients received an inappropriate ICD shock (0.5%). During follow-up, implantation of a cardiac implantable electronic device was carried out in 34.5% of patients. When comparing German patients (n = 516) to Swiss patients (n = 192), Swiss patients presented with longer wear days (70.72 ± 49.47 days versus 58.06 ± 40.45 days; p = 0.001) and a higher ICD implantation rate compared to German patients (48.4% versus 29.3%; p = 0.001), although LVEF at follow-up was similar between both groups. Young age is a negative independent predictor for the compliance in this large registry. The most common indication for WCD was non-ischemic cardiomyopathy followed by ischemic cardiomyopathy. The compliance rate was generally high with a decrease of wear hours per day at younger age. Slight differences were found between Swiss and German patients, which might be related to differences in mentality for ICD implantation.

## Introduction

Sudden cardiac death (SCD) can potentially be prevented using the wearable cardioverter defibrillator (WCD). A bevy of indications have been highlighted for the use of WCD e.g. for secondary or primary prevention of patients with ischemic (ICM) or non-ischemic cardiomyopathies (NICM) or in patients after removal of infected implantable cardioverter defibrillators (ICDs)^1^ or in patients post myocarditis, peripartum cardiomyopathy and takotsubo syndrome^26–29^.

One of the main parameters for risk stratification for SCD is the left ventricular ejection fraction (LVEF ≤ 35%)^2,3^. Up to date, data have shown that patients with a severely reduced LVEF and heart failure are at high risk of SCD^4–6^. Several data sets have shown that patients with newly diagnosed heart failure caused by ICM or NICM may present with an improvement of LVEF subsequent to optimal medical treatment (OMT). Therefore, current guidelines support a strategy of waiting for at least 6 weeks to 3 months after the initial diagnosis of highly reduced LVEF depending on the underlying etiology^7,8^. Data of the IRIS and DINAMIT trials showed that early ICD implantation after MI does not improve the survival rates^9,10^. Moreover, recently published data debated a prolonged period of OMT and a prolonged WCD use before ICD implantation in patients with newly diagnosed NICM and ICM^30^. Prolonged WCD use and OMT may allow avoiding over-use of ICDs^13^.

Although ICD implantation is feasible in young patients, a multitude of device-related complications including inappropriate shocks, infections, lead failure and lead dysfunction, which increase over time, have been reported, which may potentially be avoided by unnecessary ICD implantation.

Recently published data of the VEST trial^14^ showed that WCD do not significantly impact the outcome, measured by a composite endpoint after up to 90 days of use in patients with MI and moderate to severe left ventricular dysfunction compared to controls. Although a significant reduction in all-cause mortality (secondary endpoint), a safe use of WCD and a very low rate of inappropriate shocks in the WCD group were documented, the published data does not convincingly support the systematic use of WCD in this population.

In the present study, we investigated 708 consecutive patients from four hospitals in Germany and Switzerland. Long-term follow-up data over a mean time of 2.5 years was collected. We aimed to investigate the risk of ventricular tachyarrhythmias and the success rate of termination of these by WCD use.

## Methods

### Patient recruitment

We included 708 patients at four hospitals (University Mannheim, Frankfurt University Hospital, the Heart Center Leipzig and the University Hospital Zurich). Patients were included between 04/2012 and 03/2019. All patients received OMT. Patients were fitted with a ZOLL Life Vest System. The study was approved by the local ethics committee (2019-840-R and BASEC 2017-01044) and conforms to the 1975 Declaration of Helsinki. An informed consent was waived by the ethics committee to the retrospective character of the study. All methods were performed in accordance with the relevant guidelines and regulations by including a statement in the “Methods” section.

### The wearable cardioverter-defibrillator (WCD)

The WCD ZOLL Life Vest ™system (Pittsburgh, USA) and programmed data have been recently described in depth^15^. Different points were taken into consideration for programming including the underlying heart disease and electrocardiographic patterns. In general, for older patients the ventricular tachycardia (VT) zone was programmed at a heart rate of 150–190 bpm with a VT response time of 60 s and for younger patients a VT zone was programmed at a heart rate of 180–190 bpm with a VT response time also of 60 s. The ventricular fibrillation (VF) zone was programmed similarly in older and younger patients at a heart rate of 200–220 bpm with a response time of 25 s. The maximum first shock energy was 150 J with a separate episode detecting when episodes were common with a minimum delay of 3 min. Episodes were reviewed and classified by independent physicians. Episodes were separated into one of two groups, sustained VT (lasting 30 s or longer) or VF with WCD shock therapy and non-sustained VT (lasting less than 30 s) without WCD shock. Inappropriate WCD therapy was identified as non-ventricular tachyarrhythmias or non-ventricular fibrillation episode treated by an inappropriate WCD shock.

### Baseline and follow up data collection

Baseline characteristics were evaluated at each center including the indication for WCD use, the disease etiology, the initial LVEF calculated by the biplane Simpson’s method using echocardiography and/or cardiac magnetic resonance imaging (MRI). LVEF improvement was defined as an increase of the LVEF > 35% over follow-up. The optimal medical treatment (OMT) was documented in 96% of patients in the cohort. However, the dosage of used drugs was not studied systematically. In general, WCD use was suggested for 3 months regardless of the underlying heart disease. WCDs were prescribed consistent with current guidelines and the risk was estimated and individualized by treating physicians. Data on arrhythmias during follow-up were prospectively collected clinically and retrieved from the ZOLL Life Vest Network™.

After prescription WCD, patients were followed for a mean follow-up of 28 ± 35.5 months. This mean follow-up included follow-up after ICD-implantation. To estimate the need of ICD implantation, the LVEF was evaluated 3–6 months after the initial evaluation. If the LVEF was > 35% and no ventricular tachyarrhythmias were detected no ICD was implanted concomitant with current guidelines. In some cases of a relevant improvement of LVEF but nevertheless LVEF < 36%, prolongation of WCD wearing was applied. This was dependent on physician–patient decision.

OMT was achieved using the generally recommended heart failure drugs e.g. angiotensin converting-enzyme inhibitor/angiotensin receptor blocker (ACE-I/ARB), beta-blockers and mineralocorticoid receptor blocker (MRA) consistent with current heart failure guidelines^3^. Also, angiotensin receptor-neprilysin inhibitors (ARNI) were used instead of ACE-I or ARB consistent with published data and guidelines^31-36^.

Patient charts, general physicians, cardiologists, patients and family members were consulted for updating missing data.

### Statistics

Data are presented as mean ± standard deviation for continuous variables or as frequencies and percentages for categorical variables. For the comparison of continuous variables the paired-t-test was used and for the comparison of categorical variables a chi-squared test or Fisher's exact test was used.

A two-sided P value < 0.05 was considered statistically significant. IBM SPSS for Macintosh (Version 25.0. Armonk, NY: IBM Corp.) was used for statistical analyses. For predictor analysis Cox logistic regression analysis was done. Factors with a p value < 0.1 were included in a multivariable logistic regression analysis.

## Results

### Patients’ baseline characteristics

**Figure 1:**
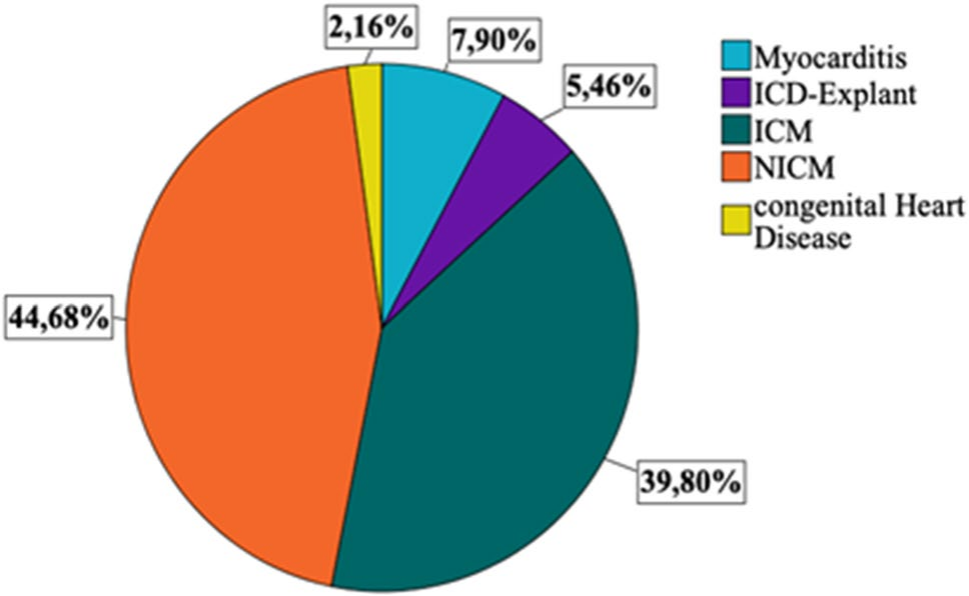
The distribution of indication for WCD us in the general cohort.

The indications for WCD use were predominantly ICM and NICM (39.9% and 44.6%). Other indications were myocarditis, ICD explantation and, in rare cases, channelopathy (Fig. 1). The reason for prescription WCD in ICM, NICM and myocarditis patients was the low LVEF. 81.8% of patients were male and 18.2% were female. The shock rate was 2.7% (n = 19), appropriate in 2.2% and inappropriate in 0.5% of patients. The mean wear time of WCD per day was 21.17 ± 4.31 h and mean wear day time was 61 days. The index LVEF was 29.66 ± 11.36% and the follow-up LVEF was 37.5 ± 12.35%. In 42.9% of patients an improvement of the LVEF was documented, with 46% of patients having a LVEF > 35% at follow-up. Implantation of a cardiac implantable electronic device was carried out in 47.7% of patients (Table 1). The satisfactory compliance rate, defined as wear hours > 20 h per day, of the whole cohort was 78.4%. The overall death rate in the cohort was 5.9%. No ventricular tachyarrhythmias were documented once the WCD was no more in use and the LVEF was improved > 35% in these patients, who did not receive an ICD.

**Table 1:** Baseline characteristics of 708 patients with WCD use.

Variables	Patients (n = 708)
Female, n (%)	129 (18.2)
Age, mean ± SD	60.68 ± 14.63
WCD wear time (h/day), mean ± SD	21.16 ± 4.34
WCD wear days, mean ± SD	61.5 ± 43.45
**WCD shocks, n (%)**	19 (2.7)
Appropriate	16 (2.2)
Inappropriate	3 (0.5)
EF baseline, mean ± SD	29.62 ± 11.20
EF follow-up, mean ± SD	37.27 ± 12.26
EF improvement, n (%)	326 (46.0)
Device implantation, n (%)	244 (34.5)
Death during follow-up, n (%)	42 (5.9)
Compliance (> 20 h/day WCD use), n (%)	555 (78.4)

### WCD data stratified by sex

**Figure 2:**
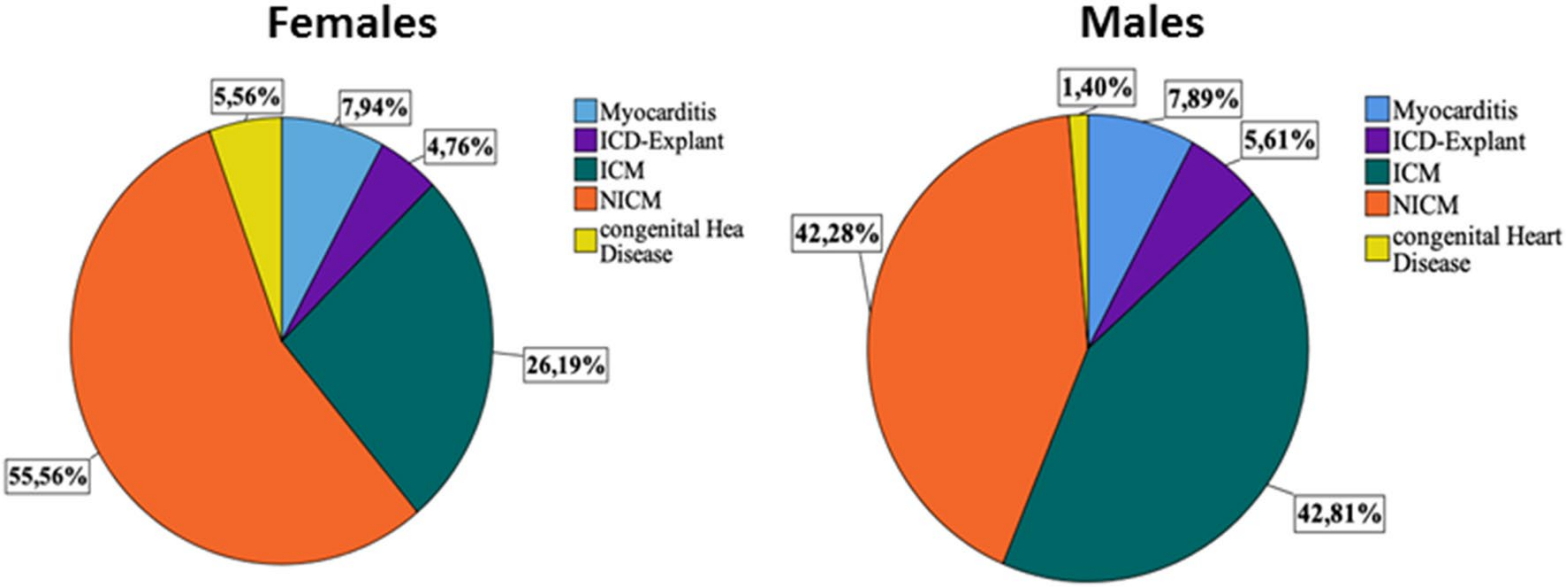
The indication for WCD use in males and females.

The mean average age and wear days were comparable in females and males at the time of WCD prescription (Table 2). The appropriate WCD shock rate was also comparable between females and males (3.8% versus 1.9%; p = 0.259). In males, the WCD use was similar in ICM and NICM. On the other hand, NICM was predominantly the cause for WCD use in females, Fig. 2. The death rate was comparable between males and females.

**Table 2:** Baseline characteristics of WCD patients stratified by sex.

	Female, n = 129	Male, n = 579	p-Value
Age, mean ± SD	61.58 ± 17.05	60.48 ± 14.04	0.441
WCD wear time (h/day), mean ± SD	21.53 ± 4.41	21.09 ± 4.29	0.282
WCD wear days, mean ± SD	56.60 ± 38.98	62.86 ± 44.36	0.140
**WCD shocks, n (%)**	5 (3.8)	14 (2.4)	0.082
Appropriate	5 (3.8)	11 (1.9)	0.259
Inappropriate	0 (0)	3 (0.5)	0.259
EF baseline, mean ± SD	30.17 ± 12.30	29.55 ± 11.14	0.549
EF follow-up, mean ± SD	38.18 ± 13.17	37.35 ± 12.17	0.486
EF improvement > 35%, n (%)	67 (51.9)	259 (44.7)	0.157
Device implantation, n (%)	41 (31.8)	203 (35.1)	0.330
Death during follow-up, n (%)	10 (7.7)	32 (5.5)	0.497
Compliance (> 20 h/day WCD use), n (%)	109 (84.5)	446 (77.0)	0.063

### WCD data stratified by etiology

Patients with ICM were significantly older compared to NICM (64.96 ± 11.09 years versus 58.62 ± 14.51 years; p = 0.001). Females were more common in the NICM group as compared to the ICM group (23.2% versus 11.7%, p = 0.001). The wear time in days and wear time in hours were comparable between both groups. The initial LVEF was significantly higher in ICM patients as compared to NICM patients, while the appropriate shock rate was similar (8 patients versus 2 patients, p = 0.371). In 44% of patients with ICM, LVEF improved during follow-up as compared to 50.8% in the NICM group. In general, all-cause mortality tended to be higher in the ICM group as compared to the NICM group (7.2% versus 4.2%, p = 0.061), (Table 3).

**Table 3:** Baseline characteristics of WCD patients stratified by cardiomyopathy etiology.

N (%)	ICM, 277 (39.7)	NICM, 315 (44.5)	p-value
Age, mean ± SD	64.96 ± 11.11	58.59 ± 14.61	0.001
Female, n (%)	33 (11.9)	70 (22.5)	0.001
WCD wear time (h/day), mean ± SD	21.32 ± 3.92	20.92 ± 4.47	0.251
WCD wear days, mean ± SD	63.22 ± 43.55	61.66 ± 43.44	0.665
**WCD Shocks, n (%)**	9 (3.2)	3 (1.0)	0.026
Appropriate	8 (2.9)	2 (0.7)	0.371
Inappropriate	1 (0.3)	1 (0.3)	0.371
EF baseline, mean ± SD	30.07 ± 8.76	26.65 ± 10.96	0.001
EF follow-up, mean ± SD	36.58 ± 10.71	36.67 ± 12.18	0.920
EF improvement > 35%, n (%)	122 (44.0)	158 (50.8)	0.111
Device implantation, n (%)	112 (40.4)	101 (32.5)	0.208
Death, n (%)	20 (7.2)	13 (4.2)	0.061
Compliance (> 20 h/day WCD use), n (%)	219 (79.1)	239 (76.8)	0.754

### Characteristics of patients treated with WCD shocks

Of the 19 patients treated with WCD shocks, 16 patients (2.2%) received an appropriate WCD shock and three patients (0.5%) inappropriate WCD shocks. Eight patients were diagnosed with ICM, two patients with NICM, three patients suffered from myocarditis and two patients received an ICD explantation after which WCD was required. The reason for a WCD in myocarditis patients was low LVEF and in one case low LVEF and non-sustained VTs.

One patient with an appropriate shock suffered from a hereditary channelopathy (Table 4). The case was a long-QT syndrome. The patient showed also a reduced LVEF at events. Since the diagnosis was not clear it was decided for a WCD prescription. Over follow-up the LVEF was normalized however the QTc was ongoing prolonged.

**Table 4:** WCD Shock Characteristics according to appropriate and inappropriate manner, n = 19.

Gender	Age	WCD Indication	LVEF at baseline	Shock appropriate	Arrhythmic episode	ICD implant	Death during 3 yr follow up	Appropriate ICD shocks
m	66	ICM	35	Yes	VT	Yes	No	Yes
f	31	Myocarditis	53	Yes	VT	Yes	No	No
m	48	ICD-Explant	30	Yes	VT	Yes	Yes	No
m	53	ICM	25	Yes	VF	Yes	No	No
m	64	ICM	35	Yes	VT	Yes	No	Yes
m	75	ICM	20	Yes	VT	Yes	No	No
f	71	ICM	30	Yes	VF	Yes	No	No
f	84	ICM	25	Yes	VT	Yes	No	No
m	64	NICM	25	Yes	VT	Yes	Yes	No
m	71	ICM	27	Yes	VF	Yes	No	Yes
m	60	NICM	13	Yes	VT	Yes	No	No
m	64	Myocarditis	40	Yes	VF	Yes	No	No
f	84	ICM	35	Yes	VT	No	No	No
m	62	Myocarditis	20	Yes	VT	Yes	No	No
f	55	ICD-Explant	55	Yes	VF	Yes	No	Yes
m	84	ICM	27	No	AFlutt	No	No	No
m	25	Channelopathy	60	Yes	VF	Yes	No	Yes
m	78	NICM	30	No	AF	Planned	Unknown	Unknown
m	64	Myocarditis	40	No	AF	No	No	No

*A.Flutt* atrial flutter, *AF* atrial fibrillation, *VT* ventricular tachycardia.

Of the 16 patients receiving an appropriate WCD shock, 5 received appropriate ICD shocks after ICD implantation during a follow-up period of 28 months.

### Characteristics of patients according to age differences

**Figure 3:**
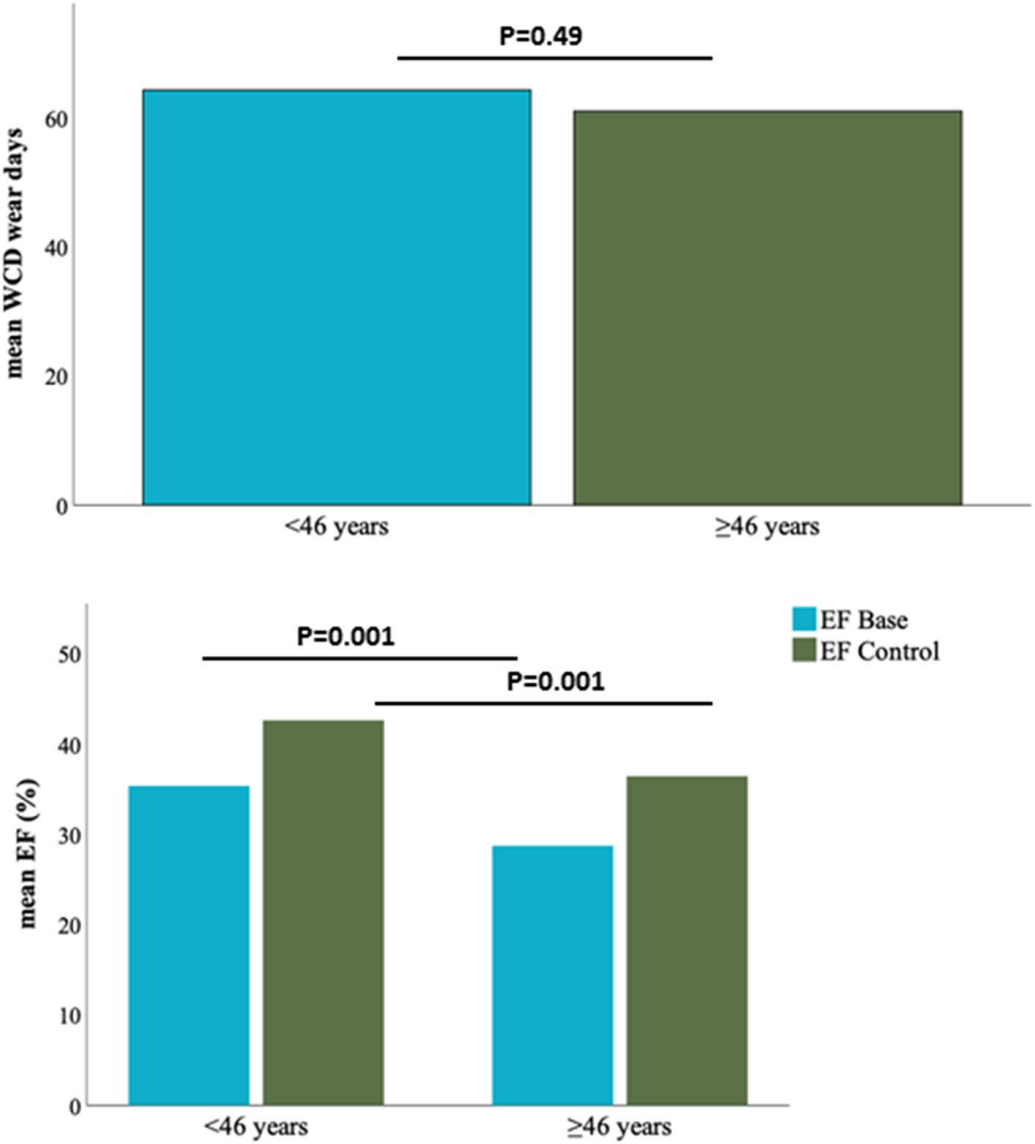
The comparison of WCD use according to age.

We separated patients into two groups: < 46 years, n = 94 and ≥ 46 years, n = 614. The average wear days were comparable between both cohorts. The initial LVEF, the follow up LVEF and the percentage of patients with an improvement of the LVEF was higher in the young cohort as compared to the old cohort, Fig. 3. The younger patients tended to have a lower compliance rate, however when setting a cut-off of 46 years, no significant difference in wear days was documented, Fig. S1.

### Characteristics of patients of the German and Swiss cohort

The cohort included 516 German patients and 192 Swiss patients. In the German cohort, 104 (20.34%), and in the Swiss cohort 25 (13.02%) patients were female; p = 0.02. German patients were older than Swiss patients (61.74 ± 15.103 years versus 57.84 ± 12.877, p = 0.002). Although Swiss patients presented with more wear days compared to German patients (70.72 ± 49.47 days versus 58.06 ± 40.45 days; p = 0.001), the wear hours per day was comparable in the Swiss cohort and the German cohort (21.29 ± 4.49 versus 20.79 ± 3.88, p = 0.179). The initial LVEF was significantly lower in German patients compared to Swiss patients (28.85 ± 10.38% versus 31.67 ± 12.97%), while the follow-up LVEF was comparable between both groups, Table 5, Fig. S2. On the other hand, the ICD implantation rate was significantly higher in Swiss patients compared to German patients (48.3% versus 29.3%; p = 0.001).

**Table 5:** Baseline characteristics of WCD patients in Germany compared to Switzerland.

	Germany, n = 516	Switzerland, n = 192	p-Value
Female, n (%)	104 (20.34)	25 (13.02)	0.029
Age, mean ± SD	61.74 ± 15.10	57.84 ± 12.88	0.002
WCD wear time (h/day), mean ± SD	21.29 ± 4.49	20.79 ± 3.88	0.179
WCD wear days, mean ± SD	58.06 ± 40.45	70.72 ± 49.47	0.001
**WCD shocks, n (%)**	16 (3.1)	3 (1.6)	0.260
Appropriate	13 (2.5)	3 (1.6)	0.414
Inappropriate	3 (0.6)	0 (0)	0.414
EF baseline, mean ± SD	28.85 ± 10.38	31.67 ± 12.97	0.003
EF follow-up, mean ± SD	37.06 ± 12.49	37.84 ± 11.63	0.455
EF improvement > 35%, n (%)	241 (46.7)	85 (44.3)	0.563
Device Implantation, n (%)	151 (29.3)	93 (48.4)	0.001
Compliant (> 20 h/day WCD wear time), n (%)	413 (80.0)	142 (74.0)	0.081

### Predictors of compliance

To understand, which are the predicting factors for a good compliance we did a Cox logistic regression. In the univariate analysis male gender (OR 0.61; 95%CI 0.36–1.02; p = 0.06) and age < 46 years (OR 0.60; 95%CI 0.37–0.97; p = 0.04) were predictors of the compliance. In the multivariable logistic regression analysis only young age (OR 0.58; 95%CI 0.36–0.95; p = 0.03) was a negative independent predictor of the compliance, Table 6.

**Table 6:** Multivariable logistic regression analysis for the compliance.

	Univariate analysis			Multivariable analysis		
	OR	95%CI	P-value	OR	95%CI	P-value
Male	0.61	0.36–1.02	**0.06**	0.60	0.35–1.00	0.05
Age < 46	0.60	0.37–0.97	**0.04**	0.58	0.36–0.95	**0.03**
NICM	0.85	0.59–1.21	0.37			
Myocarditis	1.67	0.77–3.62	0.18			
ICD explantation	0.88	0.40–1.90	0.75			
EF at baseline	1.00	0.99–1.02	0.43			
ICM	1.06	0.73–1.54	0.72			
WCD shocks	5.21	0.70–38.48	0.10			
EF improvement	1.16	0.80–1.66	0.42			

*OR* odds ratio, *EF* ejection fraction, *NICM* non-ischemic-cardiomyopathy, *WCD* wearable life-vest.

## Discussion

We analyzed real life experience with WCD in Germany and Switzerland. Patients were recruited in 4 centers and data was collected for three years. The findings of the present project are: (i) WCD use in the present cohort was required due to NICM followed by ICM, (ii) The daily wear time of the present cohort with 21.17 ± 4.31 h is high and implicates excellent patient compliance in Germany and Switzerland; (iii) The general shock rate is 2.7%, the appropriate shock rate being 2.2% and the inappropriate shock rate being low with 0.5%; iv) the younger the patients, the lower the compliance rate as presented by wear hours per day.

Sudden cardiac death is one of the main causes of death worldwide predominantly due to ICM^16^. The majority of patients suffer from ventricular tachyarrhythmias. The efficacy of WCD has been confirmed in several registries and reports^15,17–19^. The appropriate WCD shock rate in our study was 2.2%. This rate is slightly higher compared to other reported cohorts from the USA and France, which varied between 1.6 and 1.7%. Of the 16 patients treated with an appropriate WCD shock and subsequent ICD implantation, 5 (31%) patients suffered from an appropriate ICD shock during follow-up. Data from the WEARIT-II Registry and WCD France Registry showed that WCD is safe with low inappropriate shock rates of 0.5% and 0.7%, respectively, which was similar in our multicenter registry. In general, these data are consistent throughout multiple studies and confirm the safety of the WCD^20^.

Current ESC guidelines recommend that WCD may be indicated in patients with heart failure related to a decline in LVEF (≤ 35%) as a bridge solution until either recovery of LVEF, heart transplant or ICD re-implantation in case of prior device explantation or de-novo ICD implantation^1^. Overall, the presented data confirms the role of WCD in selected patients for the prevention of SCD. The DANISH trial, as one of the first trials focusing exclusively on patients with NICM, confirmed the risk of ventricular tachyarrhythmias in the chronic phase of NICM, during which primary preventive ICD implantation on the other hand did not reduce overall mortality^21^. But there was a significant interaction with age and younger patients showed a significant reduction in all-cause mortality after ICD implantation. Therefore, in NICM an individualized manner might be suggested regarding the decision on ICD implantation taking into consideration severity of underlying disease and concomitant arrhythmia risk versus age, life expectancy and risks of competing illness.

It was reported that during 12 months of follow-up, 4% of patients with WCD died and the overall death rate between ICM and NICM was comparable^22^. In the present cohort we found a mortality rate of 6.1% over a follow-up of almost 2.5 years and the mortality rate in the ICM group tended to be higher than the NICM group (Table 3). Data from a large cohort from the USA found a 3-month mortality rate of 3.6% and a 3-year mortality of 20.5%. This higher rate of mortality might be due to the heterogeneity of the USA cohort and different comorbidities compared to the present cohort^15^.

One important issue is the compliance of patients wearing the WCD. A lack of adherence to therapy (i.e. not wearing the WCD) is one of the possible causes for the lack of positive results measured in the VEST trial which included patients after acute MI^14^. Indeed, in a per-protocol analysis of the VEST trial the authors found a significant reduction of all-cause and arrhythmic mortality^23^. These results however have to be interpreted with caution due to the inherent risk of bias when performing per-protocol analyses. Nevertheless, patients should be educated and regularly reminded of the importance of wearing the WCD for the entire prescription period. In the present cohort, a high compliance rate with 78.4% of patients wearing the WCD > 20 h per day were achieved. Different reports discussed different predictors of this adherence to wear the WCD. Recently published data reported that a younger age is associated with a less compliance^20^. Our data shows a different compliance rate for wearing the WCD from 16 to 46 years old. After age of 45 years no significant difference regarding the compliance was documented. We evaluated predictors of the compliance and found young age is a negative independent predictor of the compliance. This is might be related to the character of young patients with underestimating their disease and/or absence of symptoms.

Data from other reports showed that the average hours of use per day were 20.7 in males and 21.4 in females (p = 0.001) with a repetitive WCD shock rate in females suffering from ICM^24,25^. In the present cohort we found similar average wear hours in females compared to males. In general, our data present a high compliance for WCD use consistent with other reports.

Beside the VEST trial which had a low daily compliance of 14 ± 9.3 h, other reports, albeit from registry data, present a high compliance of patients. Therefore, different factors should be taken into consideration when WCD is prescribed such as sufficient education and training of the patient. Additionally, close follow-ups are required to avoid failure rates regarding to incompliance.

### Limitations of the study

The present study has several limitations. Firstly, the use of magnetic resonance imaging for the outcome of patients and risk stratification of WCD cohort is not considered. In addition, the prescription was based on physician decision and individualized risk factors. Thirdly, a detailed cause of death was not evaluated in a systematic manner in patients. Finally, a further evaluation of predictors for appropriate WCD shock rate according to the cause of NICM form is not possible related to the low WCD shock rate, which should be analyzed in an expanded cohort.

## Conclusion

The most common indication for WCD was NICM followed by ICM. The compliance rate was generally high with a decrease of wear hours per day at younger age. Slight differences were found between Swiss and German patients, which might be related to differences in mentality for ICD implantation.

## Supplementary Information

Supplementary Figures.
