# Supplementary material for: Real life experience with the wearable cardioverter-defibrillator in an international multicenter Registry
Source: Sci Rep. 2022 Feb 25;12:3203. doi: 10.1038/s41598-022-06007-y (PMC8881447; doi:10.1038/s41598-022-06007-y)

**Figure S1:** The comparison of WCD compliance between <46 years and ≥46 years

**Figure S2:** The distribution of WCD use cause in Germany compared to Switzerland


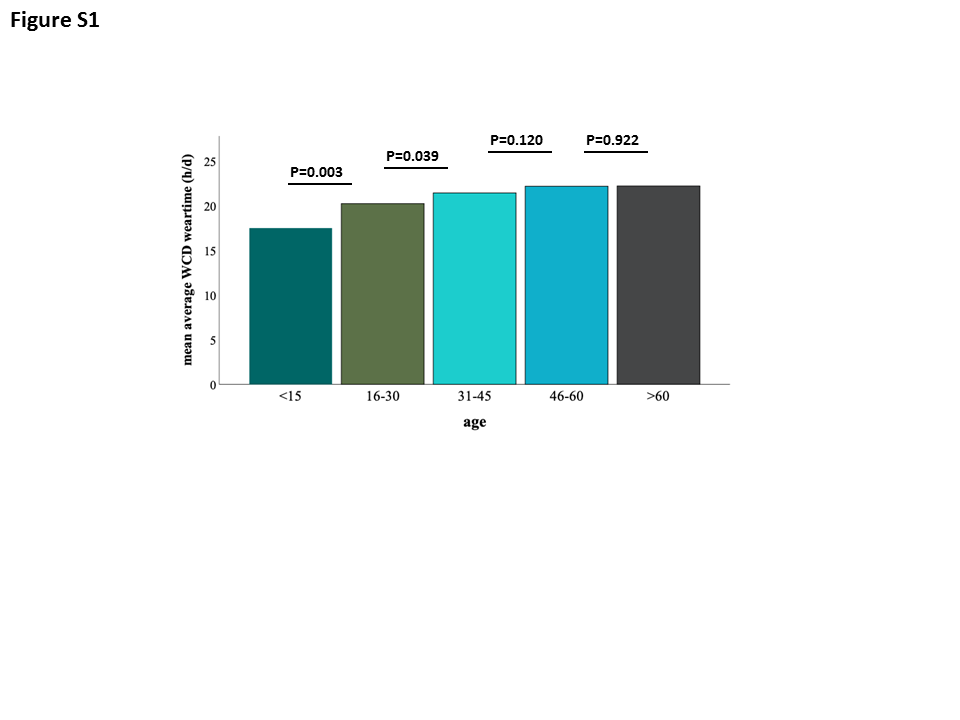


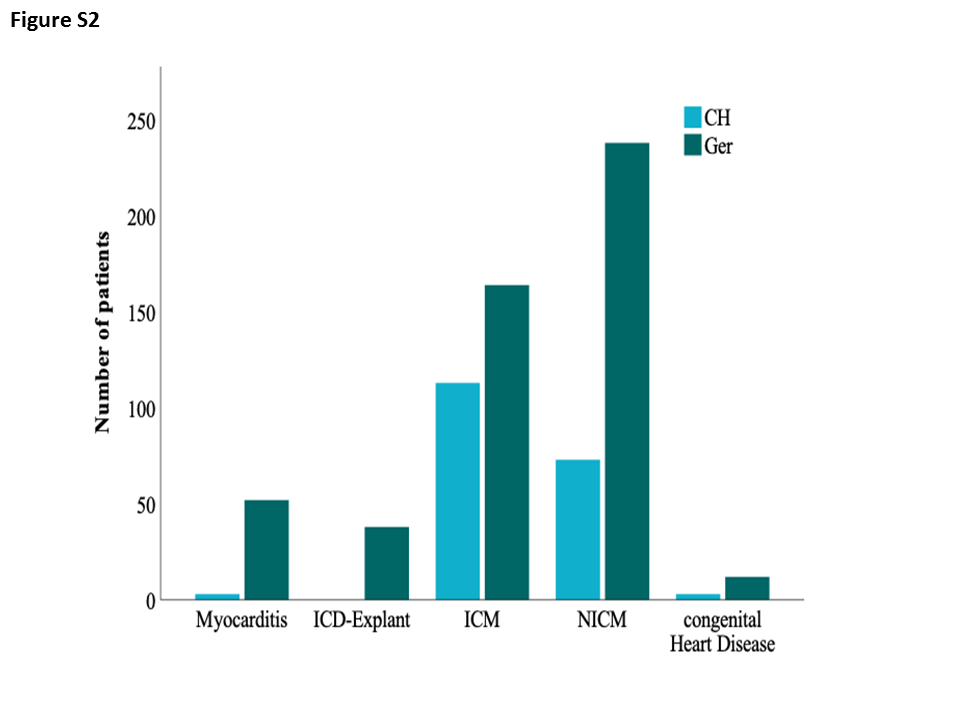

Supplement: Supplementary file 1 — Supplementary Figures. [file 41598_2022_6007_MOESM1_ESM.docx]
